# Acute urticaria as a side effect of the Mirena® (levonorgestrel-releasing intrauterine system): a case report

**DOI:** 10.1186/1756-0500-7-209

**Published:** 2014-04-03

**Authors:** Xiangjuan Chen, Xueqing Wu, Haiyan Zhu

**Affiliations:** 1 Department of Obstetrics and Gynecology, The First Affiliated Hospital of Wenzhou Medical College, 2# Fuxue Road, Wenzhou 325000, People’s Republic of China

**Keywords:** Mirena®, Allergic reactions

## Abstract

**Background:**

The levonorgestrel intrauterine system, Mirena®, is widely used for contraception and the treatment of idiopathic menorrhagia. Here, we reported one case of acute urticaria following Mirena® implantation to increase the awareness of possible adverse side effects associated with Mirena®.

**Case presentation:**

The case presented is a 27-year-old Chinese woman who received Mirena® implantation for her adenomyosis and menorrhagia. The operation was successful and the patient did not experience any discomfort during the operation. However, she developed acute urticaria on her entire body accompanied with pruritic, slight left lower quadrant pain, and slight dizziness two hours after the operation. The patient was recommended to have the Mirena® removed immediately, and she took 10 mg oral methylprednisolone and 5 mg desloratadine tablet daily for five days. Her urticaria resolved and did not recur.

**Conclusion:**

The patient’s acute urticaria seems to have been associated with the Mirena® levonorgestrel intrauterine system implantation, since she had no history of allergic reactions to materials used during the operation such as plastic, metal, alcohol, medications, and povidone-iodine.

## Background

The levonorgestrel intrauterine system (LNG-IUS), Mirena®, is a commonly used contraceptive device in clinical practice. In addition to providing effective contraception, Mirena® is also frequently used to treat idiopathic menorrhagia and protect the uterus from endometrial hyperplasia in estrogen replacement therapy [1]. Adverse side effects associated with Mirena® implantation include acne, menstrual disorder, pelvic pain, and ovarian cysts. Most of these symptoms can resolve spontaneously within several months [1-3]. Adverse skin reactions to Mirena® are rare. Karri *et al*. reported one case of severe seborrhoeic dermatitis, which is thought to associate with the levonorgestrel in Mirena® [4]. Here, we reported one case of acute urticaria following Mirena® implantation. The patient was advised to have the Mirena® removed immediately and took 10 mg oral methylprednisolone and 5 mg desloratadine tablet daily for five days. Her urticaria then resolved and did not recur.

## Case presentation

A 27-year-old Chinese woman gravida 1 para 1 was treated with Mirena® implantation for adenomyosis and menorrhagia in our hospital in September 2012. The patient was in good general health and had regular menstrual cycles. She had a caesarean section to deliver a baby boy in 2003. Six months after the delivery, she received a triangular-shaped metal intrauterine device (IUD) insertion for contraception. The IUD was then removed two months later due to unbearable vaginal spotting. The patient had a history of moderate alcohol consumption but no history of allergic reactions to plastic, metal, alcohol, medications, and povidone-iodine. Her routine gynecological examination before the operation appeared normal.

**Figure 1:**
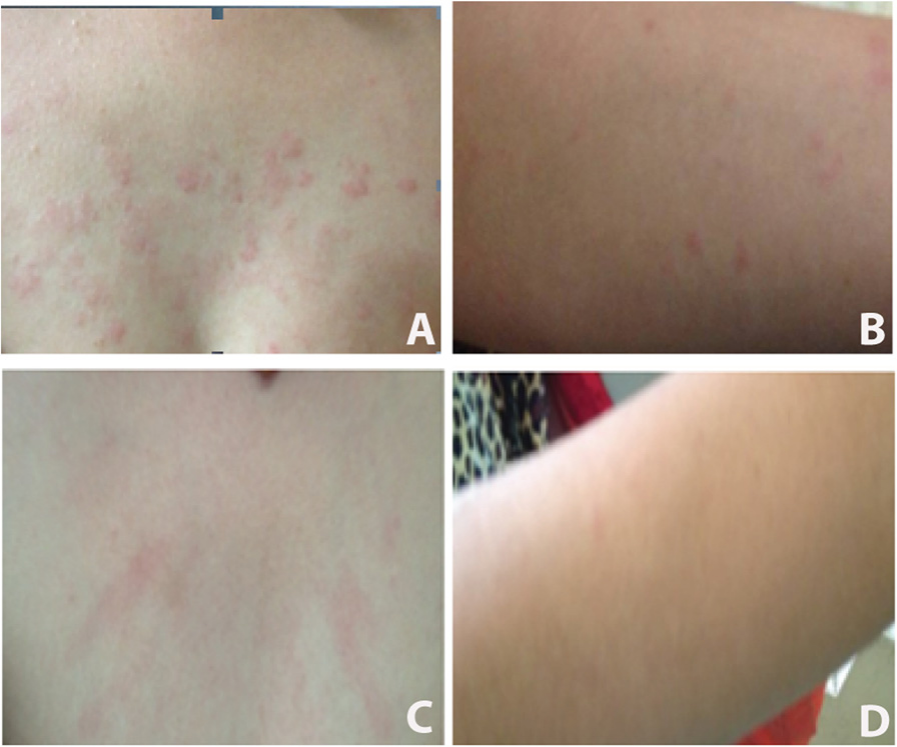
**Images of erythematous skin rash on the patients.** Eruption of erythematous skin rash on the patient’s chest **(A)** and right forearm **(B)** two hours after the Mirena® implantation. The urticaria on the patient’s chest **(C)** and right forearm **(D)** was significantly relieved three days after the medication of oral methylprednisolone and desloratadine tablet.

A Mirena® LNG-IUS was inserted into her uterine cavity according to the manufacturer’s instruction. The patient did not complain any discomfort during the operation. The operation was successful. However, 2 hours after the operation, the patient developed an erythematous skin rash on her body. The lesion first appeared around her umbilical area, then on her chest, face, legs, arms, and feet. The rash covered her entire body in a short time (Figure 1A and B). Other symptoms accompanied with the development of the rash included pruritic, slight left lower quadrant pain, and slight dizziness. The patient did not have fever, nausea, and vomiting. Her vital signs appeared normal and stable. Her gynecological examination did not showed any significant abnormality. Her vulva development was normal. She was sexually active and had no prior vaginal delivery. Her genital skin and mucosa did not exhibit any rash and congestion. Small amount of bloody discharge was present in her vagina. Her cervix was smooth. Her uterus was anteverted, slightly enlarged, and soft. She did not feel tenderness around uterus area. Her ovaries and ovarian attachments did not show any obvious abnormality.

The diagnosis from a dermatologist was acute urticaria possibly caused by allergic reactions. The patient was advised to have the Mirena® removed immediately. After the Mirena® was removed, her pruritic was slightly relieved. The patient then took 10 mg oral methylprednisolone and 5 mg desloratadine tablet daily for five days. The patient came back to follow-up visit three days after the operation. Her abdominal pain and pruritic resolved completely, her skin condition also improved significantly (Figure 1C and D). The medication was discontinued five days after the operation. Her urticaria subsided and did not recur.

## Discussion

The most commonly recognized side effects associated with Mirena® are vaginal bleeding or spotting, amenorrhea, and benign ovarian cysts [1-3]. Although adverse skin reactions to Mirena® are rarely seen, a few cases of skin condition have been reported to associate with Mirena® implantation. Karri *et al*. presented a case of severe seborrhoeic dermatitis [4]. Levonorgestrel is the hormonal component of the Mirena® LNG-IUS. The patient’s seborrhoeic dermatitis is thought to be caused by the levonorgestrel released from the Mirena®, because her skin condition was improved quickly after the removal of the Mirena®. Pereira *et al*. reported a case of autoimmune progesterone dermatitis. In their report, the patient developed itchy skin rash 24 hours after a Mirena® IUS insertion. Her symptoms were initially alleviated by local steroid and oral antihistamines, but resolved eventually by the removal of the Mirena® IUS [5].

In this report, this was the first case of acute urticaria possibly associated with Mirena® implantation we encountered in our hospital. This patient developed a rash eruption on her entire body 2 hours after the implantation. The lesions carry the typical characteristics of urticaria, such as intensive pruritus and becoming blanched completely with pressure. To determine the possible association between the lesions and the Mirena® implantation, we carefully examined the patient’s condition and medical history. The patient had no food intake before, during, and after the operation, eliminating food related allergic reactions. She was not on any regular medication and had no new medicines before the operation. Thus, drug allergies might be excluded. In addition, she had no history of allergic reactions to any medication or the material used during the operation including plastic, metal, alcohol, and povidone-iodine. The patient had no recent or chronic infections or other medical conditions. The only medical procedure she was exposed to was the Mirena® implantation. Based on these facts, we concluded that her urticarial condition was most likely due to the Mirena® implantation. Similar to the reports from Karri *et al*. and Pereira *et al*., the acute urticaria in this case might also be associated with the levonorgestrel released from the Mirena® LNG-IUS.

Although the rash is likely associated with Mirena® implantation, limitations regarding the diagnosis should not be ignored. Our diagnosis of Mirena® -induced acute urticaria is mainly based on the fact that the rash appeared 2 hours after the implantation and the patient did not have previous allergic reactions to the materials used during the operation. However, since we did not examine the serum levels of immunoglobulins indicating possible allergic reactions to the materials used during the operation, such as plastic, metal, alcohol, and povidone-iodine, the possibility that these things might contribute to the development of acute urticaria in this patient cannot be excluded completely. In addition, it has been known that emotional stress can induce acute urticaria as well. According to the patient’s medical history, in 2003, she had caesarean section and triangular-shaped metal IUD insertion for contraception, which have a similar operating procedure as Mirena® LNG-IUS implantation. Although the patient was not recorded to exhibit any abnormal reactions to the two previous operations, she might experience unusual anxiety and fear during the Mirena® implantation. Thus, we cannot completely rule out the possibility that the acute urticaria in this case might relate to the operation procedure but not directly associate with the Mirena® LNG-IUS device.

The levonorgestrel released from Mirena® is at a higher concentration in the uterus than that in the systemic circulation. Thus, the side effects of Mirena® are usually local and mild. We reported this case of acute urticaria possibly associated with Mirena® implantation to raise the awareness of the side effects of Mirena® and emphasize the prompt treatment for the adverse reactions.

## Conclusion

We reported one case of acute urticaria possibly associated with Mirena® implantation. The patient’s adverse skin reaction might relate to the levonorgestrel released from the Mirena® LNG-IUS. Her symptoms were improved by oral steroid and antihistamines and removal of the Mirena®, and did not recur after the medication was discontinued.

## Patient consent

Written informed consent was obtained from the patient for publication of this case report and any accompanying images. A copy of the written consent is available for review by the Editor-in-Chief of this journal.

## Competing interests

The authors declare that they have no competing interests.

## Authors’ contributions

XC carried out the study and drafted the manuscript. XW helped to collect the data. HZ initiated the study and helped to draft the manuscript. All authors read and approved the final manuscript.
